# GPR120 induces regulatory dendritic cells by inhibiting HK2-dependent glycolysis to alleviate fulminant hepatic failure

**DOI:** 10.1038/s41419-021-04394-0

**Published:** 2021-12-16

**Authors:** Hongshuang Yu, Wanlin Yang, Jiefang Huang, Xiang Miao, Bei Wang, Xiaohui Ren, Yuting Gu, Qiwei Wang, Xinyuan Ding, Xin Guo, Fengtao Qian, Yanyun Zhang, Huanbai Xu, Leizhen Zheng, Min Jin

**Affiliations:** 1 Clinical Research Centre, The Affiliated Changshu Hospital of Xuzhou Medical University, Changshu, China; 2 Shanghai Institute of Nutrition and Health, Shanghai Institutes for Biological Sciences, University of Chinese Academy of Sciences, Chinese Academy of Sciences, Shanghai, China; 3 Children’s Hospital of Soochow University, Institutes for Translational Medicine, State Key Laboratory of Radiation Medicine and Protection, Key Laboratory of Stem Cells and Medical Biomaterials of Jiangsu Province, Medical College of Soochow University, Soochow University, Suzhou, Jiangsu China; 4 Department of Stomatology, Renji Hospital, School of Medicine, Shanghai Jiao Tong University, Shanghai, China; 5 Department of Endocrinology and Metabolism, Shanghai General Hospital, Shanghai Jiao Tong University School of Medicine, Shanghai, China; 6 Department of Oncology, Xinhua Hospital Affiliated to Shanghai Jiao Tong University School of Medicine, Shanghai, China

**Keywords:** Inflammation, Hepatitis

## Abstract

Fulminant hepatic failure (FHF) is a potentially fatal liver disease that is associated with intrahepatic infiltration of inflammatory cells. As the receptor of polyunsaturated long chain fatty acids, GPR120 can regulate cell differentiation, proliferation, metabolism, and immune response. However, whether GPR120 is involved in FHF remains unknown. Using *Propionibacterium acnes* (*P. acnes*)-primed, LPS-induced FHF in mice, we found that interference with GPR120 activity using pharmacological agonist attenuated the severity of the liver injury and mortality of FHF in mice, while a lack of GPR120 exacerbated the disease. GPR120 activation potently alleviated FHF and led to decreased T helper (Th) 1 cell response and expansion of regulatory T cells (Tregs). Interestingly, GPR120 agonist didn’t directly target T cells, but dramatically induced a distinct population of CD11c^+^MHC II^low^CD80^low^CD86^low^ regulatory DCs in the livers of FHF mice. GPR120 was found to restrict HIF-1α-dependent glycolysis. The augmented HIF-1α stabilization caused by GPR120 antagonism or deletion could be attenuated by the inhibition of ERK or by the activation of AMPK. Through the analysis of the clinical FHF, we further confirmed the activation of GPR120 was negatively associated with the severity in patients. Our findings indicated that GPR120 activation has therapeutic potential in FHF. Strategies to target GPR120 using agonists or free fatty acids (FFAs) may represent a novel approach to FHF treatment.

## Introduction

Fulminant hepatic failure (FHF) is a complicated and devastating disorder with high morbidity and mortality due to the abrupt loss of hepatic function [1]. Increases in intrahepatic infiltration of inflammatory cells are mainly associated with the pathogenesis of FHF [1]. Although the pathogenesis of FHF has been extensively investigated, there is no proper therapeutic strategies for this disease, leading to high mortality if there is no supportive management or liver transplantation [2–5]. Thus, effective anti-inflammatory and immunosuppressive measures are urgently needed for FHF therapy, and the critical molecular mechanisms governing the hyper-inflammation associated with FHF need to be further clarified.

Dendritic cells (DCs) serve as the key mediators of the initiation and regulate both innate and adaptive immune responses. In contrast to the well-known role in effectively priming T cells by conventional DCs (cDCs), regulatory DCs serve to induce immune tolerance and have been used successfully to prevent the onset of inflammatory diseases [6]. Published studies have shown that treatment of DCs with certain immunosuppressive drugs and molecules leads to the generation of regulatory DCs, which show downregulation of the major histocompatibility complex class II (MHC II) or costimulatory molecules [7]. Regulatory DCs could produce immunosuppressive cytokines, and possess tolerogenic properties including the inhibition of T-cell activation and induction of regulatory T cells (Tregs) [7]. To develop an effective strategy for intervention of T-cell-mediated immune disorders, we have previously established the modified DCs with moderately high levels of MHC molecules that are defective in the expression of costimulatory molecules induced by mesenchymal stem cells, which we therefore designated as regulatory DCs [8].

Priming mice by the injection of heat-killed *Propionibacterium acnes* (*P. acnes*) followed by lipopolysaccharide (LPS) treatment is one of the most commonly used animal models of FHF [8–10]. The pathogenesis in this model can be divided into two different phases with regard to the immune responses [11]. In the priming phase, activated Kupffer cells (KCs) secrete chemokines such as macrophage inflammatory protein-1α to recruit a subset of CD11c^+^B220^−^ DC precursors from the blood to the liver, which is an initiating event and a prerequisite for liver injury in this model [9, 12]. DC precursors differentiate into mature DCs and are activated by *P. acnes* and inflammatory cytokines secreted by KCs. Activated DCs then migrate into hepatic lymph nodes and activate *P. acnes*-specific CD4^+^ T cells [10, 12]. Subsequently, the T cells, macrophages, and DCs that accumulate in liver produce a variety of cytokines, promoting the formation of granulomas and hepatocyte damage after the injection of LPS [13]. Therefore, inhibition of inflammatory infiltration and immune responses evoked by DCs is beneficial for treatment of FHF.

G-protein-coupled receptor 120 (GPR120) has been implicated in the pathogenesis of cancers, diabetes, and inflammatory diseases [14, 15]. GPR120 can regulate many cellular functions, including inflammation, differentiation, metabolism, and proliferation [16, 17]. Several long-chain fatty acids, which are GPR120 ligands, also prevent inflammatory and metabolic disorders [18, 19]. Although GPR120 has been reported to mediate hepatoprotective effects against hepatic ischemia reperfusion injury, alcoholic liver disease, and nonalcoholic fatty liver disease [20–23], the role of GPR120 in acute inflammatory liver injury remains largely unknown.

In this study, we found that GPR120 activation could effectively attenuate disease severity and decrease mortality in a mouse model of *P. acnes*-primed and LPS-induced FHF. GPR120 deletion, on the contrary, exacerbated the disease. Importantly, we demonstrated that GPR120 ameliorated disease progression by inducing regulatory DCs in the liver, which then restrained T helper (Th) 1 cell immune responses and promoted the differentiation of Tregs. Mechanistically, GPR120 was found to induce regulatory DCs by restraining hypoxia-inducible factor-1α (HIF-1α) and subsequently glycolysis via the ERK and AMPK pathways. GPR120 activation was also shown to be negatively associated with the severity of FHF in patients. Taken together, our results indicate that GPR120 plays a protective role in *P. acnes*-induced liver injury and might be a potential clinical target for the treatment of FHF.

## Materials and methods

### Animals

C57BL/6 mice were purchased from the Shanghai Laboratory Animal Center of the Chinese Academy of Sciences and maintained under specific pathogen-free conditions in the vivarium of Shanghai Institute of Nutrition and Health, Chinese Academy of Sciences. Constitutive GPR120-knockout (GPR120^−/−^) mice were purchased from Shanghai Bioray Laboratory (Shanghai, China). All animal experiments were performed according to the guide of the Institutional Animal Care and Use Ethics Committee of the Shanghai Institutes for Biological Sciences, Chinese Academy of Sciences, and complied with the Guide for the Care and Use of Laboratory Animals published by the U.S. National Institutes of Health.

### Human sample collection

The collection of human subjects was approved by the Human Ethics Committee, Ruijin Hospital, Shanghai Jiao Tong University School of Medicine. Written consent was obtained from each subject. The subjects included healthy controls (*n* = 9) and hepatitis B virus (HBV) -related FHF patients (*n* = 16) who were admitted to the Department of Infectious Diseases, Ruijin Hospital, Shanghai, China from June 2020 to August 2020 and their evaluation was based on the criteria of the Asian Pacific Association for the Study of the Liver. Among these HBV-FHF patients, three out of four patients were cirrhotic. And ten of them suffered HBV for more than 30 years, three for 23 years, two for 10 years, and one for five years. Under steady-state conditions, human peripheral blood (PB) DCs are defined as cells that lack lineage (Lin) markers (i.e., CD3, CD15, CD19, CD14, CD20, and CD56) and constitutively express HLA-DR (referred to as Lin^−^HLA-DR^+^ pan-DCs) [24]. Human PB DCs are broadly categorized into two major subsets: cDCs and plasmacytoid DCs (pDCs). cDCs are characterized as Lin^−^HLA-DR^+^CD11c^+^ cells, whereas pDCs are Lin^−^HLA-DR^+^CD11c^−^CD123^high^ cells [24, 25]. Both subsets of DCs mediate T cell immune responses but have differences in Toll-like receptor (TLR) expression, inflammatory cytokine secretion, and antigen-presenting abilities [25].

### *P. acnes*-induced liver injury

Female wild-type (WT) and GPR120^−/−^ C57BL/6 mice (8–10 weeks old) were injected with 1 mg of heat-killed *P. acnes* via the tail vein. For the treatment experiments, TUG891 (10 mg/kg, Tocris, Bristol, UK) was administered intraperitoneally (i.p.) every two days after *P. acnes* priming. For survival analysis, the mice were administered an intravenous injection of 1 μg of LPS seven days after *P. acnes* priming (*n* = 7 per experimental group, a total of 28 mice were used in the experiment). On day 7, mice were sacrificed in each group (*n* = 7 per experimental group, a total of 28 mice were used in the experiment). Approximately 0.8–1 mL of blood was obtained by cardiac puncture under ether anesthesia, and liver and spleen specimens were sampled. Hepatocellular damage was determined by serum aminotransferase levels. For in vivo proliferation assays, the mice were injected i.p. with BrdU (Sigma-Aldrich, St Louis, MO, USA) one day before sacrifice (*n* = 7 per experimental group, a total of 28 mice were used in the experiment). All animal experiments were performed with the approval of the Ethics Committee for Animal Experimentation of the Shanghai Institute of Nutrition and Health, Chinese Academy of Sciences. All efforts were made to minimize animals’ suffering.

### Cell preparation

To isolate mononuclear cells (MNCs) from the liver, liver samples from mice were minced and pressed through a 70-μm nylon mesh (BD Falcon, Tewksbury, MA, USA). The cell suspension was treated with Percoll (GE Healthcare, Chicago, IL, USA) and centrifuged to remove liver parenchymal cells. The pellets were treated with red blood cell lysis solution and then washed and resuspended.

To isolate MNCs from the spleen, spleen samples from mice were minced and pressed through a 70-μm nylon mesh (BD Falcon). The cell suspension was added on Ficoll (Lymphoprep^TM^, Norway), and MNCs were isolated by density gradient centrifugation. The white layer of cells was collected.

### DC maturation and activation

Bone marrow cells (BMCs) were isolated and cultured in the presence of granulocyte-macrophage colony-stimulating factor (GM-CSF; 10 ng/mL) and IL-4 (5 ng/mL) (both from PeproTech, Cranbury, NJ, USA) for five days to induce immature DCs (immature DC culture phase). Cells were further incubated with GM-CSF (10 ng/mL) and TNF-α (50 ng/mL, PeproTech) on type I collagen-coated plates for three more days (mature DC induction phase). LPS (0.1 μg/mL) was added on day 7 to further promote DC maturation. BAY 87-2243, MK2206, LY294002, GSK621, SCH772984 (all from Selleck Chemicals, Houston, TX, USA), TUG891 (Tocris) or AH7614 (Tocris) was added into the medium as figure legends described.

### Mixed lymphocyte response (MLR)

Splenic MNCs isolated from allogeneic mice were incubated in complete medium at 37 °C for 60 minutes. Non-adherent cells were collected and sorted by magnetic cell sorting to obtain highly purified CD4^+^ T cells. DCs (0.5 × 10^5^ cells/well) from WT C57BL/6 mice underwent different treatments and were then cocultured with CD4^+^ T cells (5 × 10^5^ cells/well) isolated from WT BALB/c mice in Roswell Park Memorial Institute (RPMI) 1640 medium for 1–3 days. WT DCs were stimulated with TUG891 with or without AH7614 pretreatment. In some experiments, an inhibitor or agonist was added to the DC culture medium. The expression of CD25 and CD69 on CD4^+^ T cells was analyzed by flow cytometry the next day.

### Clinical diagnostic parameters

The following clinical characteristics of all enrolled participants were collected: alanine aminotransferase (ALT), aspartate aminotransferase (AST), prothrombin time (PT), total bilirubin (TBil), creatinine and the international normalized ratio (INR). The model for end-stage liver disease (MELD) was calculated as follows: MELD = 3.8 × ln (bilirubin [mg/dL]) + 11.2 × ln (international normalized ratio) + 9.6 × ln (creatinine [mg/dL]) + 6.4 × (etiology: 0 if cholestatic or alcoholic, one otherwise).

### Quantitative real-time PCR

Total RNA was extracted from cells using TRIzol reagent (Sigma-Aldrich) and then reverse-transcribed by a Reverse Transcription System (Takara, Tokyo, Japan). Quantitative PCR was performed using FastStart Universal SYBR Green master kit (Roche, Natley, New Jersey, USA) on a ViiA 7 Real-Time PCR System (Applied Biosystems). The reaction protocol used was 95 °C for 5 min, followed by 35 cycles of 95 °C for 15 s, 60 °C for 60 s, and 72 °C for 5 min. The expression of individual genes was normalized to the mRNA level of β-actin. The gene-specific PCR primers (all for mouse genes) are shown in Table S1.

### Immunoblot analysis

Cells were lysed with ice-cold RIPA buffer (Beyotime, China) containing a protease inhibitor cocktail and phosphatase inhibitor cocktail (Merck Millipore, Ithaca, NY, USA). The lysates were fractionated by SDS-PAGE and analyzed by western blotting with specific antibodies against GAPDH (Cell Signaling Technology, Danvers, MA, USA, CST Cat# 2118, RRID: AB_561053), G6PD (CST Cat# 8866, RRID: AB_10827744), HK2 (CST Cat# 2867, RRID: AB_2232946), PDH (CST Cat# 2784, RRID: AB_2162928), LDHA (CST Cat# 2012, RRID: AB_2137173), HIF-1α (CST Cat# 36169, RRID: AB_2799095), phosphorylated and total AMPKα (CST Cat# 2535, RRID: AB_331250 and CST Cat# 5831, RRID: AB_10622186), phosphorylated and total ERK1/2 (CST Cat# 4370, RRID: AB_2315112 and CST Cat# 4695, RRID: AB_390779), phosphorylated and total AKT (CST Cat# 13038, RRID:AB_2629447; CST Cat# 4058, RRID: AB_331168, and CST Cat# 4685, RRID: AB_2225340).

### Histological analysis

Mouse liver tissue samples were fixed in 4% paraformaldehyde, embedded in paraffin after dehydration and then sectioned at a thickness of 5 μm. Paraffin sections were deparaffinized and rehydrated in a graded alcohol series followed by hematoxylin and eosin staining. Liver nodules and infiltration of lymphocytes were shown to evaluate liver damage. Semiquantitative analysis of the status of liver inflammation was performed in a blinded manner as previously described. Briefly, the H&E-stained liver slides were scored by a pathologist in a “blinded fashion” to determine the degree of inflammation as follows: 0 = no infiltration, 1 = minimal/slight infiltration, 2 = moderate infiltration, and 3 = severe infiltration.

### TUNEL Assay

Collected liver tissues were routinely embedded into OCT and then were stored at −80 °C. For TUNEL assay, apoptotic cells were detected using a TUNEL Apoptosis Detection Kit (Alexa Fluor 488, Cat. No. 40307ES60, YEASEN Biotechnology) according to the manufacturer’s instructions.

### Flow cytometry staining

Livers and spleens were collected from different treatments of FHF mice after euthanasia. MNCs were isolated from the livers or spleens. The LIVE/DEAD™ Fixable Dead Cell Stain Kit (Invitrogen, Carlsbad, CA, USA) was used to evaluate the viability of mammalian cells. Meanwhile, MNCs were stained with anti-CD4 (Biolegend Cat# 100451, RRID: AB_2564591), anti-CD8 (Biolegend Cat# 100725, RRID: AB_493425), anti-CD25 (eBioscience Cat# 17-0251-82, RRID: AB_469366), anti-CD69 (eBioscience Cat# 45-0691-82, RRID: AB_1210703), anti-CD44 (eBioscience Cat# 12-0441-83, RRID: AB_465665), anti-CD62L (Biolegend Cat# 104406, RRID: AB_313093), anti-F4/80 (eBioscience Cat# 17-4801-82, RRID: AB_2784648), anti-CD11c (BD Biosciences Cat# 553801, RRID: AB_395060), anti-CD80 (BD Biosciences Cat# 553769, RRID: AB_395039), anti-CD86 (BD Biosciences Cat# 553692, RRID: AB_394994), anti-BV220 (eBioscience Cat# 12-0452-83, RRID: AB_465672), anti-MHC II (Biolegend Cat# 107631, RRID: AB_10900075), anti-CD103 (BD Biosciences Cat# 557495, RRID: AB_396732) and CD45 (BioLegend Cat# 103132, RRID: AB_893340) antibodies. For Th1 cell and Treg analysis, cells were stained for surface markers, permeabilized with the intracellular fixation and permeabilization buffer set (eBioscience Cat# 88-8824-00), and then stained with anti-IFN-γ (Biolegend Cat# 505825, RRID: AB_1595591), anti-TNF-α (eBioscience Cat# 11-7321-82, RRID: AB_465418) and anti-Foxp3 (eBioscience Cat# 17-5773-82, RRID: AB_469457) antibodies. For human DC analysis, cells were stained with anti-CD3 (BioLegend Cat# 300429, RRID: AB_893301), anti-CD14 (eBioscience Cat# 45-0149-42, RRID: AB_1518736), anti-CD15 (Biolegend Cat# 323019. RRID: AB_893259), anti-CD19 (Biolegend Cat# 302207, RRID: AB_314237), anti-CD20 (eBioscience Cat# 12-0209-41, RRID: AB_2572553), anti-CD56 (Biolegend Cat# 318305, RRID: AB_604093), anti-HLA-DR (eBioscience Cat# 25-9956-42, RRID: AB_1582284), anti-CD11c (BD Biosciences Cat# 564079, RRID: AB_2725779), anti-CD123 (BD Biosciences Cat# 563362, RRID: AB_2738158) and anti-GPR120 (NOVUS Cat# NBP1-00858, RRID: AB_1503311). We used combination of CD3, CD14, and CD15 as Lin1 cocktail, combination of CD19, CD20, and CD56 as Lin2 cocktail. The samples were analyzed by flow cytometry using CytoFLEX LX (Beckman Coulter).

### Glucose uptake

For glucose uptake assessment, the media was replaced with DMEM (Dulbecco’s modified Eagle’s medium) /F-12 containing the fluorescent D-glucose derivative 2-NBDG (30 μM, Invitrogen) and incubated for 2 h at 37 °C. Glucose uptake was stopped by washing the cells three times with ice-cold phosphate-buffered saline (PBS). Cells were then collected for flow cytometry analysis as soon as possible. Glucose uptake was determined by measuring the fluorescence of 2-NBDG in the cells, which typically displays excitation/emission maxima of ~465/540 nm. The data were analyzed with FlowJo software.

### Free fatty acid mass spectrometry analysis

For fatty acid extraction, the plasma was collected from human peripheral blood. For fatty acid measurement, 2 mL of methanol and 4 mL of chloroform were added to plasma samples and shaken. Then, 2 mL of double-distilled water and Na_2_SO_4_ were added to the tubes and mixed for 2 min. The lower clear liquid was transferred to a new, clean centrifuge tube, and nitrogen was used to remove the air from the tubes. N-hexane, C19:0 internal standard and KOH/methanol were added and vortexed for 2 min. After incubation for 30 min at 37 °C, 2 mL of double-distilled water was added. Nitrogen was used to remove the air from the tubes, and then 1 mL of hexane was added to the tubes. The vortexed mixtures were centrifuged, and the upper phase was used for fatty acid analysis by Gas chromatography-mass spectrometry (GC–MS).

### Statistical analysis

All data are shown as the mean ± SEM from at least three independent experiments. Significant differences were evaluated using one-way ANOVA or the Mann–Whitney U test with GraphPad Prism (version 7.0, GraphPad Software) and Statistical Package for Social Science software (version 22.0, SPSS). The Log-rank test was used for survival analysis. The correlation between two variables was assessed using Spearman’s rank correlation test. Values of *p* less than 0.05 were considered significant. Sample sizes of all experiments were predetermined by calculations derived from our experience. No sample was excluded from the analyses. Animals were not randomly assigned during collection, but the strain, sex, and age of the mice were the same, and the data analysis was single masked. Investigators were not blinded to the group allocation during the experiment and outcome assessment. The number of replicates and statistical method were indicated in each figure legend.

## Results

### GPR120 knockout exacerbated the severity of bacteria-induced liver injury in mice, while GPR120 activation ameliorated disease severity

**Fig. 1:**
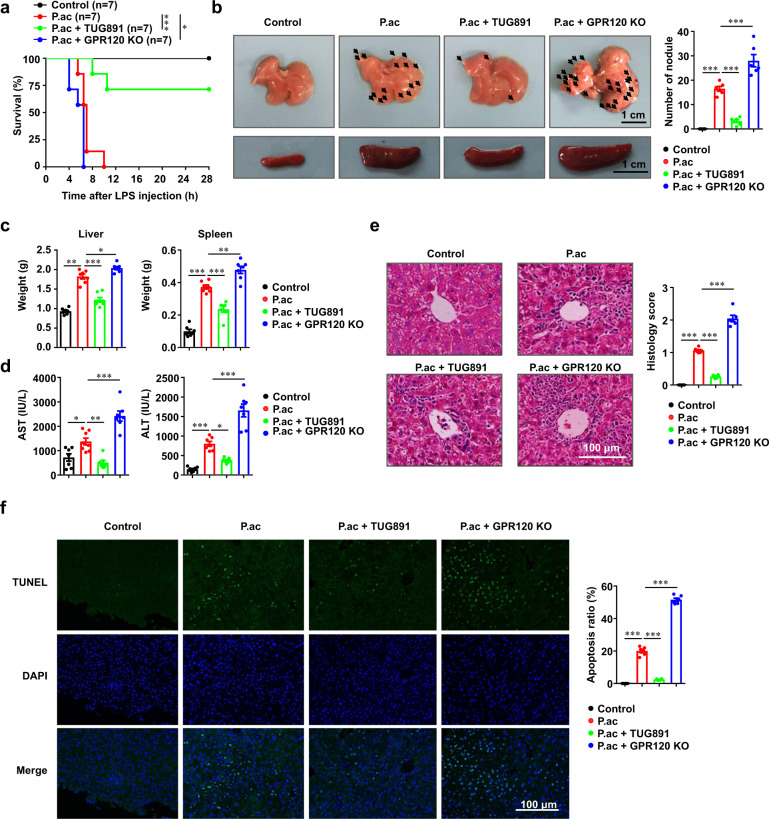
Lack of GPR120 exacerbated, whereas activation of GPR120 ameliorated, the severity of bacteria-induced liver injury. Wild-type (WT) and GPR120^−/−^ (GPR120 KO) mice were injected with *P. acnes* (P.ac) suspended in 100 μL of PBS. Vehicle or TUG891 (10 mg/kg) was administered i.p. to WT primed mice on days 0, 2, 4, and 6 (*n* = 7 mice per group). **a** One microgram of LPS in 100 μL of PBS was injected into all *P. acnes*-primed mice on day 7 to aggravate FHF. Cumulative survival rates were analyzed. **b** Liver and spleen tissues were isolated and photographed. Representative images from one of three experiments were shown. **c** The weights of livers and spleens from the four groups was measured. **d** Serum was collected on day 7, and the levels of ALT and AST were measured. **e** Liver tissues were sectioned for histological examination and semiquantitative analysis of inflammatory conditions in the liver were shown. Scale bar, 100 μm. **f** Frozen sections of livers were used for TUNEL staining. Scale bar, 100 μm. The results are representative of three to six independent experiments and presented as the mean ± SEM. Significant differences were analyzed by Log-rank test (**a**), One-way ANOVA (**b**–**f**). *p < 0.05, **p < 0.01, ***p < 0.001.

We first investigated the role of GPR120 in survival of mice with bacteria-induced liver injury. As shown in Fig. 1a, while approximately 43% of *P. acnes*-primed WT mice died within 6.5 h in response to subsequent LPS injection, all GPR120^−/−^ mice died within 6.5 h. In contrast, when treated with GPR120 agonist TUG891, 71% of the *P. acnes*-primed mice survived the initial 12 h and beyond. Furthermore, there were more hepatic granulomas in GPR120^−/−^ mice than in WT mice after priming with *P. acnes*, while TUG891-treated WT mice exhibited the lowest (Fig. 1b). Spleen enlargement also occurred in proportion to liver damage (Fig. 1b). As shown in Fig. 1c, the livers and spleens from *P. acnes*-primed mice were heavier than those from control-group mice. TUG891 significantly reduced the weight of livers and spleens, while GPR120 deletion increased the weight of livers and spleens in *P. acnes*-primed mice (Fig. 1c). These results were consistent with the dramatic decrease and increase of ALT and AST levels in the serum of TUG891-treated WT mice and in GPR120^−/−^ mice, respectively, after they were primed (Fig. 1d). In addition, larger nodules, more severe lymphocyte infiltration, and the increased levels of TUNEL^+^ cells were observed in the liver tissues on day 7 post-*P*. acnes priming in GPR120^−/−^ mice, while TUG891-treated mice showed fewer nodules, less infiltration of lymphocytes, and lower level of hepatocyte apoptosis than *P. acnes*-primed mice (Figs. 1e and f). Taken together, these data demonstrated that the lack of GPR120 exacerbated liver injury, while TUG891 treatment effectively attenuated the severity of bacteria-induced liver injury and improved the survival rate of FHF mice.

### GPR120 inhibited CD4^+^ T cell responses in bacteria-induced liver injury mice

**Fig. 2:**
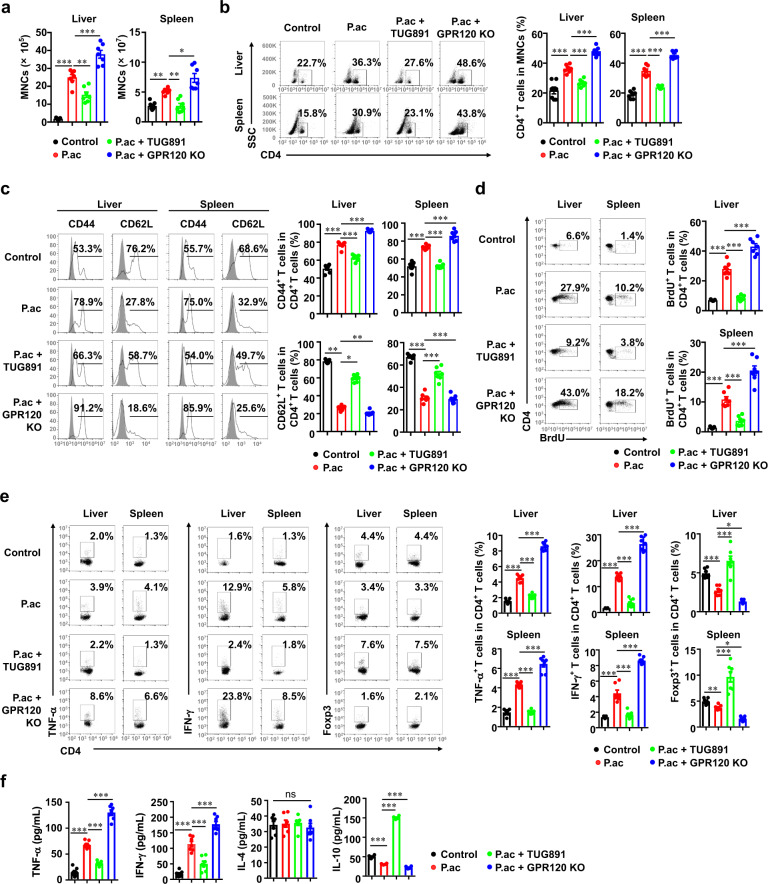
GPR120 reduced CD4^+^ T cell responses in mice with liver injury. Wild-type (WT) and GPR120^−/−^ (GPR120 KO) mice were injected with *P. acnes* (P.ac). Vehicle or TUG891 (10 mg/kg) was administered i.p. to WT mice on days 0, 2, 4, and 6 after *P. acnes* injection. Livers and spleens were isolated on day 7 (*n* = 7 mice per group). **a** Absolute numbers of total MNCs in livers and spleens were analyzed by flow cytometry. **b** Percentages of CD4^+^ T cells in total MNCs in livers and spleens were analyzed by flow cytometry. **c** The expression levels of CD44 and CD62L in CD4^+^ T cells in the livers and spleens were analyzed by flow cytometry. **d** On day 6 after priming, BrdU was injected i.p. into the mice. MNCs were isolated from livers and spleens the next day. Cells were stained for CD4 and BrdU. The frequencies of CD4^+^BrdU^+^ T cells in the livers and spleens were determined by flow cytometry. **e** The percentages of TNF-α^+^, IFN-γ^+^, and Foxp3^+^ cells in CD4^+^ T cells were assessed by intracellular staining and flow cytometry. **f** The levels of serum TNF-α, IFN-γ, IL-4 and IL-10 were measured by enzyme-linked immunosorbent assay. The results are representative of three to six independent experiments and presented as the mean ± SEM. Significant differences were analyzed by One-way ANOVA. *p < 0.05, **p < 0.01, ***p < 0.001, ns no significance.

It has been reported that CD4^+^ T cell-mediated inflammation plays a major role in *P. acnes*-induced liver injury [26–28]. We therefore investigated T cell infiltration in liver and spleen after *P*. *acnes* priming. After treatment with TUG891, the absolute number of MNCs in the liver and spleen was reduced, while GPR120^−/−^ mice had significantly increased numbers of MNCs (Fig. 2a). The percentages of CD4^+^ T cells in the liver and spleen in the different groups of mice showed a similar trend (Fig. 2b). Next, we focused on the functional change in CD4^+^ T cells. Decreased expression of CD44 and increased expression of CD62L were observed in both the liver and spleen of TUG891-treated mice (Fig. 2c), suggesting that TUG891 suppressed CD4^+^ T cell activation in primed mice. Moreover, there was considerably fewer bromodeoxyuridine (BrdU)-positive CD4^+^ T cells in the liver and spleen of TUG891-treated mice than in control-group mice, indicating that TUG891 inhibited the proliferation of CD4^+^ T cells (Fig. 2d). However, in GPR120^−/−^ mice, CD4^+^ T cells had increased expression of CD44 and reduced expression of CD62L (Fig. 2c). More BrdU-positive CD4^+^ T cells were also observed in the liver and spleen of GPR120^−/−^ mice after *P*. *acnes* priming (Fig. 2d). Taken together, these results suggested that GPR120 negatively regulated CD4^+^ T cell activation and proliferation in bacteria-induced liver injury.

Th1 cells have been identified as central players in the pathogenesis of *P. acnes*-induced liver injury [29]. Thus, the levels of the Th1 cytokines tumor necrosis factor (TNF)-α and interferon (IFN)-γ were measured. As shown in Fig. 2e, TUG891 treatment potently inhibited the production of IFN-γ and TNF-α in hepatic and splenic CD4^+^ T cells, while GPR120 deletion further increased their production (Fig. 2e). Moreover, the administration of TUG891 significantly increased the percentage of CD4^+^Foxp3^+^ Tregs, while the GPR120 deletion reduced the percentage of these cells (Fig. 2e). Then the levels of the cytokines TNF-α, IFN-γ, IL-4, and IL-10 in the serum were tested, and the results showed levels of inflammatory cytokines were decreased but the level of anti-inflammatory cytokine, IL-10, was increased after TUG891 treatment (Fig. 2f). These data demonstrated that TUG891 suppressed Th1 cells but promoted Tregs during liver injury.

**Fig. 3:**
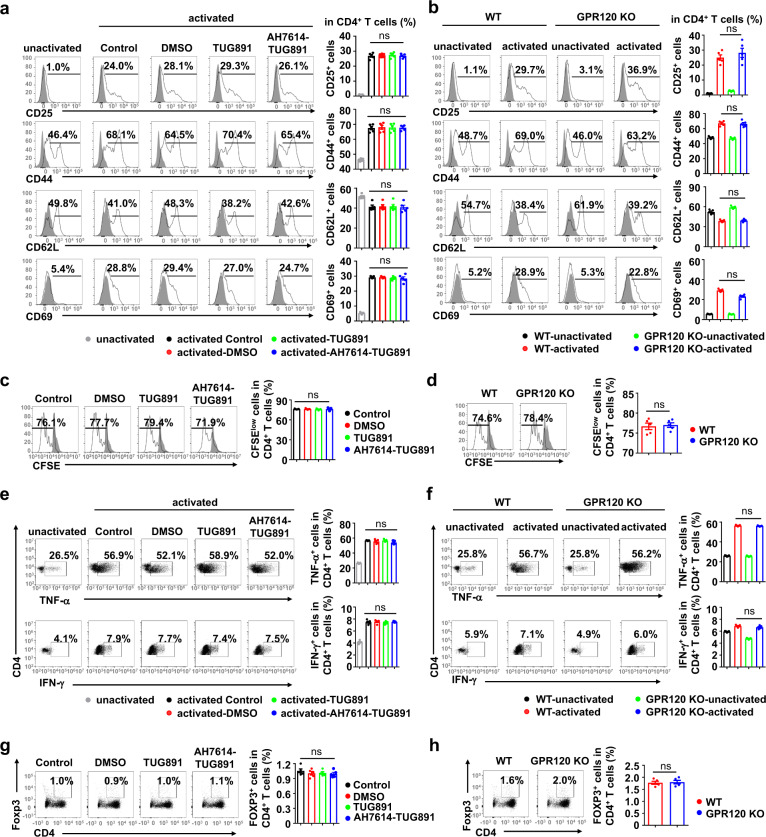
GPR120 had no effects on the activation, proliferation, and differentiation of CD4^+^ T cells in vitro. T cells were isolated from the spleens of wild-type (WT) mice and GPR120^−/−^ (GPR120 KO) mice. T cells were cultured (2 × 10^5^ cells/well) in the presence of anti-CD3 and anti-CD28 antibodies. T cells from WT mice were treated with DMSO, TUG891 (20 μM) with or without AH7614 (20 μM). **a**, **b** The expression levels of CD25, CD44, CD62L, and CD69 on CD4^+^ T cells were analyzed by flow cytometry. **c**, **d** Frequencies of the proliferating (CFSE^low^) CD4^+^ T cell proliferation in response to the indicated treatments were measured by flow cytometry. **e**, **f** CD4^+^ T cells were stained for the surface marker CD4 and intracellular expression of TNF-α and IFN-γ on day 5 and analyzed by flow cytometry. **g**, **h** CD4^+^ T cells were stained for the surface marker CD4 and intracellular expression of Foxp3 on day 5 and analyzed by flow cytometry. The results are representative of three independent experiments and presented as the mean ± SEM. Significant differences were analyzed by One-way ANOVA. ns no significance.

To explore the function of GPR120 in CD4^+^ T cell immunity in vitro, the GPR120 agonist TUG891, and antagonist AH7614 were then individually used. Interestingly, activation and proliferation were not remarkably different among control-group, TUG891-treated, AH7614-TUG891-treated, and GPR120^−/−^ CD4^+^ T cells (Figs. 3a–d). Intracellular staining of TNF-α, IFN-γ, and Foxp3 also revealed no significant difference among activated CD4^+^ T cells with regard to GPR120 status (Figs. 3e–h). These results indicated that the effect of GPR120 on CD4^+^ T cells may be indirect.

### GPR120 induced regulatory DCs to inhibit CD4^+^ T cell responses

**Fig. 4:**
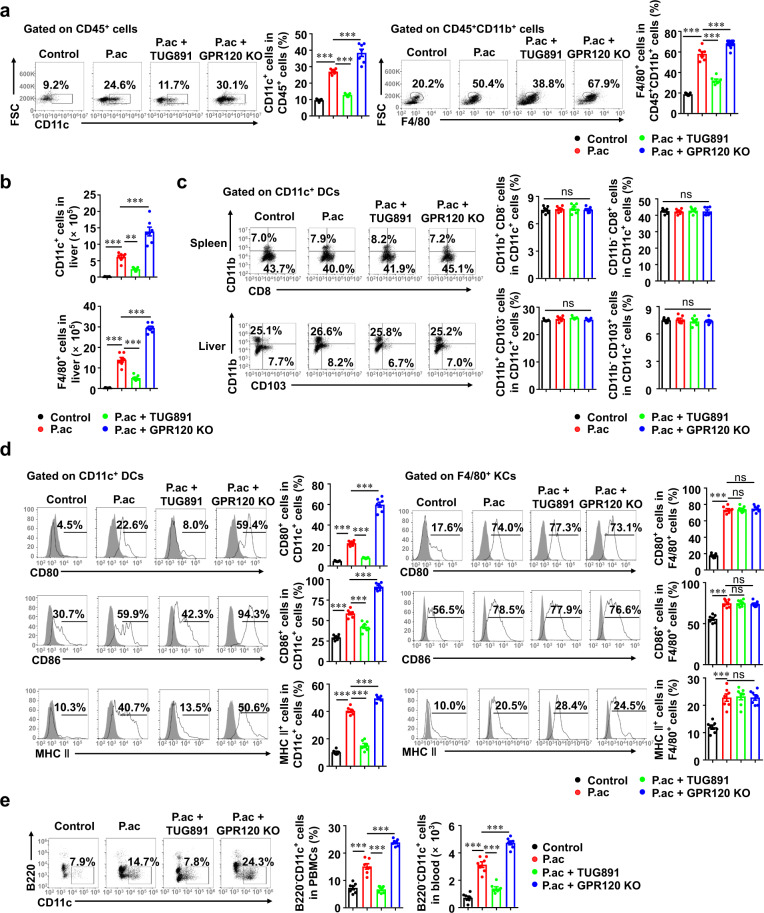
GPR120 regulated the activation and maturation of DCs, but had no effects on macrophage activation. Wild-type (WT) and GPR120^−/−^ (GPR120 KO) mice were injected with *P. acnes* (P.ac). Vehicle or TUG891 (10 mg/kg) was administered i.p. to WT mice on days 0, 2, 4, and 6 after *P. acnes* injection. Livers and peripheral blood were isolated from the mice (*n* = 7 mice per group). **a** Percentages of CD11c^+^ DCs in liver CD45^+^ cells and F4/80^+^ KCs in liver CD45^+^CD11b^+^ cells were analyzed by flow cytometry. **b** Absolute numbers of CD11c^+^ DCs and F4/80^+^ KCs in liver MNCs were analyzed by flow cytometry. **c** The percentages of splenic and liver DCs (CD11b and CD8 in the spleen) and (CD103 and CD11b in the liver) were analyzed by flow cytometry. **d** Levels of CD80, CD86, and MHC II on F4/80^+^ KCs and CD11c^+^ DCs in livers were analyzed by flow cytometry. **e** The percentages and numbers of CD11c^+^B220^−^ DC precursors in the peripheral blood MNCs from each group were analyzed by flow cytometry. The results are representative of three independent experiments and presented as the mean ± SEM. Significant differences were analyzed by One-way ANOVA. **p < 0.01, ***p < 0.001, ns no significance.

We next determined whether antigen-presenting cells, such as KCs and DCs, were directly targeted by GPR120 to protect against FHF. The percentage and number of CD11c^+^ DCs and F4/80^+^ KCs in the liver of primed mice administered with TUG891 were decreased, while they were increased in GPR120^−/−^ mice after priming (Figs. 4a and b). TUG891 treatment or GPR120 deletion did not affect splenic and hepatic DC subsets among FHF mice (Fig. 4c) and the expression of MHC II or costimulatory molecules (e.g., CD80 and CD86) in F4/80^+^ KCs compared with those of *P. acnes*-primed mice (Fig. 4d). However, TUG891 treatment and GPR120 deletion respectively reduced and increased CD80, CD86, and MHC II expression in CD11c^+^ DCs (Fig. 4d). Thus, DCs, but not KCs, might be responsible for GPR120-mediated alleviation of FHF.

**Fig. 5:**
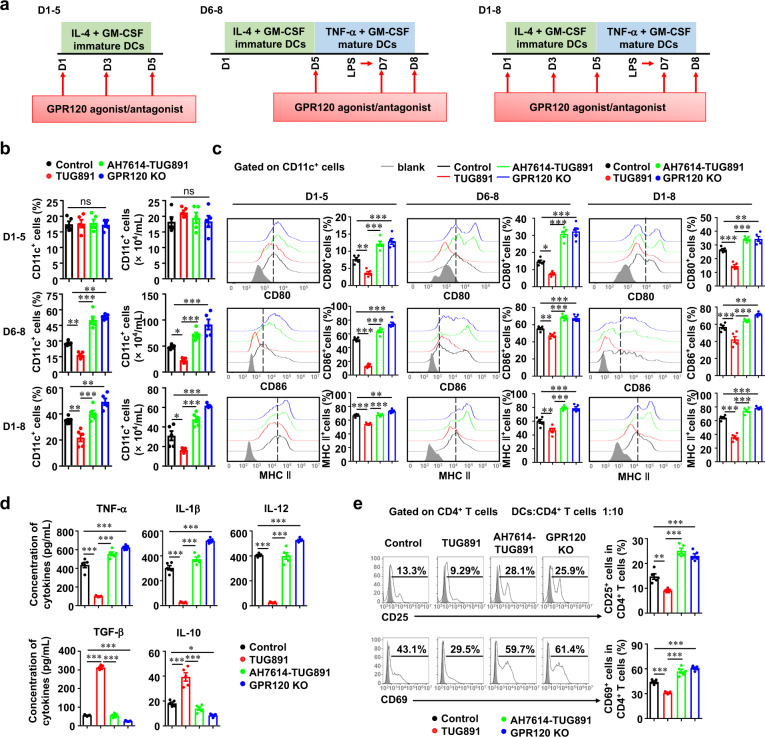
GPR120 induced the generation of regulatory DCs. **a** Schema of the induction of DC maturation with GPR120 agonist or antagonist treatment in vitro. BMCs from wild-type (WT) mice were isolated and incubated with GM-CSF (10 ng/mL) plus IL-4 (5 ng/mL) for 5 days, and GM-CSF (10 ng/mL) plus TNF-α (50 ng/mL) for three additional days. LPS (0.1 μg/mL) was added on day 7. Cells were stimulated with TUG891 (20 μM) with or without AH7614 (20 μM) pretreatment on days 1–5, days 6–8, or days 1–8. **b** The percentages and numbers of CD11c^+^ DCs were analyzed by flow cytometry on day 5 and day 8. **c** Cells were stained for CD11c, CD80, CD86, and MHC II and analyzed by flow cytometry on day 5 and day 8. **d** The levels of TNF-α, IL-1β, IL-12, TGF-β, and IL-10 were measured by enzyme-linked immunosorbent assay. **e** CD4^+^ T cells (5 × 10^5^ cells/well) from the spleens of WT BALB/c mice were cocultured with WT DCs or GPR120^−/−^ DCs (0.5 × 10^5^ cells/well) from C57BL/6 mice. WT DCs were treated with TUG891 (20 μM) with or without AH7614 (20 μM) pretreatment. The expression of CD25 and CD69 on CD4^+^ T cells was analyzed by flow cytometry the next day. The results are representative of three independent experiments and presented as the mean ± SEM. Significant differences were analyzed by One-way ANOVA. *p < 0.05, **p < 0.01, ***p < 0.001, ns no significance.

We previously reported that the recruitment of CD11c^+^B220^−^ DC precursors from blood to liver was an initial event in *P. acnes*-induced liver injury model [9]. The precursors gradually differentiated into functional, mature DCs and elicited liver immune responses and liver injury [8]. As shown in Fig. 4e, TUG891 treatment decreased the percentage and number of DC precursors in the blood, while GPR120 deletion had an opposite effect. Then, we examined whether GPR120 altered the potential of DC precursors to differentiate into mature DCs. In vitro experiments showed that TUG891 inhibited the percentage and number of CD11c^+^ DCs, while AH7614 and GPR120 deletion increased those during DC maturation induction period but not during the immature culture phase (Figs. 5a and b), suggesting that GPR120 could inhibit the differentiation of DC precursors into mature DCs. In addition, TUG891 treatment promoted DCs to acquire the phenotype of CD11c^+^CD80^low^CD86^low^MHC II^low^, which could be defined as regulatory DCs (Fig. 5c). Consistently, AH7614 pretreatment and GPR120 deletion enabled the activation of DCs during the maturation induction as well as the immature culture period (Fig. 5c). Furthermore, TUG891-treated DCs produced lower levels of proinflammatory cytokines, including TNF-α, IL-1β, and IL-12, than control-group DCs but higher levels of anti-inflammatory cytokines TGF-β and IL-10 (Fig. 5d). These data indicated that TUG891-treated DCs possessed the function of immune suppression, which were distinct from mature or immature DCs.

Then, TUG891-treated DCs or DCs from GPR120^−/−^ mice were co-cultured with WT CD4^+^ T cells to further confirm the role of GPR120 in DC function. As expected, the expression of CD25 and CD69 on CD4^+^ T cells was decreased when the cells were co-cultured with TUG891-treated DCs but increased when the cells were co-cultured with AH7614 and TUG891-pretreated or GPR120^−/−^ DCs (Fig. 5e), suggesting that the effect of GPR120 on T cell immune response was attributed to the generation of regulatory DCs.

### GPR120 induced regulatory DCs by inhibiting glycolysis

**Fig. 6:**
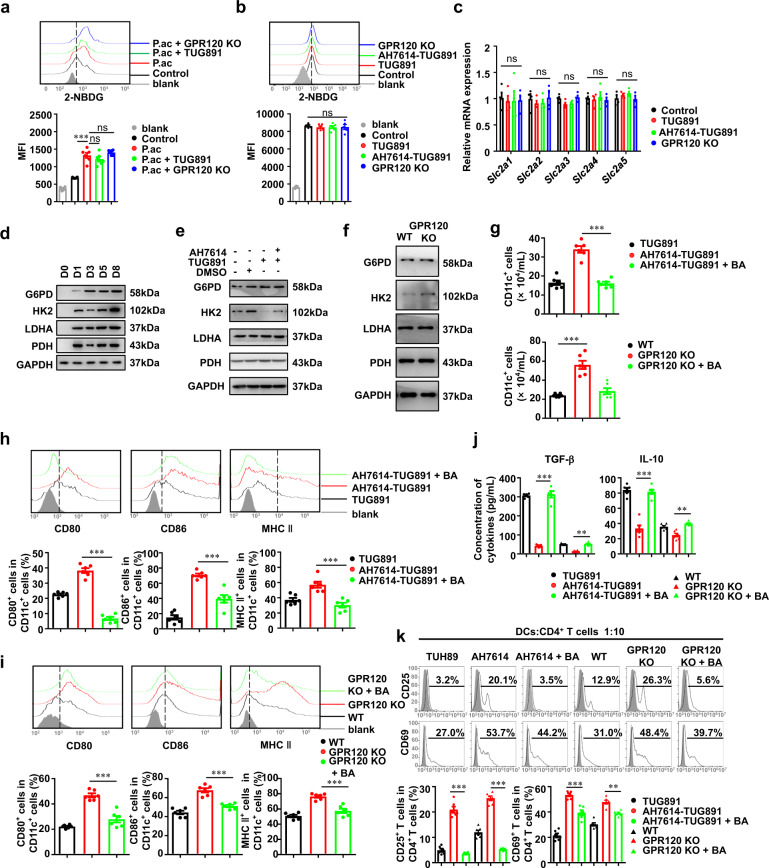
GPR120 activation induced regulatory DCs by inhibiting glycolysis. **a** Wild-type (WT) and GPR120^−/−^ (GPR120 KO) mice were injected with *P. acnes* (P.ac). Vehicle or TUG891 (10 mg/kg) was administered i.p. to WT mice on days 0, 2, 4, and 6 after *P. acnes* injection (*n* = 7 mice per group). Glucose uptake of CD11c^+^ DCs in mouse livers was measured by 2-NBDG assay. Mean fluorescence intensity (MFI) of 2-NBDG was shown. **b**–**f** BMCs from WT and GPR120^−/−^ mice were isolated and cultured to induce maturation. TUG891 (20 μM) with or without AH7614 (20 μM) pretreatment was added to the medium of BMCs from WT every 2 days. LPS (0.1 μg/mL) was added on day 7. CD11c^+^ cells were separated using magnetic cell sorting microbeads. **b** 2-NBDG was added, and glucose uptake was determined by flow cytometry. MFI of 2-NBDG was shown. **c** Quantitative real-time PCR was used to determine the mRNA expression of *Slc2a1-5* in DCs on day 8. **d**, **f** Protein expression of G6PD, HK2, LDHA, and PDH in DCs was determined by immunoblot analysis during DC maturation or with GPR120 interference on day 8. **g**–**k** The glycolysis inhibitor bromopyruvic acid (BA, 5 μM) was added to the DC induction medium. **g** The numbers of CD11c^+^ DCs among the cultured cells were analyzed by flow cytometry on day 8. **h**, **i** The expression of CD80, CD86, and MHC II of CD11c^+^ DCs was analyzed by flow cytometry on day 8. **j** Levels of TGF-β and IL-10 were measured by enzyme-linked immunosorbent assay. **k** CD4^+^ T cells were cocultured with DCs that underwent the indicated treatments for 16 h. CD25 and CD69 expression in CD4^+^ T cells was analyzed by flow cytometry. The results are representative of three to six independent experiments and presented as the mean ± SEM. Significant differences were analyzed by One-way ANOVA. **p < 0.01, ***p < 0.001, ns no significance.

Nutrients and biosynthesis play important roles in promoting the activation and maturation of DCs, for which glucose metabolism plays important roles [30]. Glucose uptake is the first step in glucose utilization and is mediated by glucose transporters encoded by the *Slc2a* family genes [31]. Therefore, the ability of DCs to take up glucose was measured in *P. acnes*-primed mice. We found that neither GPR120 activation nor GPR120 deletion had an effect on the glucose uptake of hepatic CD11c^+^ DCs (Fig. 6a). Measurement of glucose uptake in WT DCs stimulated with TUG891/AH7614 in vitro and GPR120^−/−^ DCs produced similar results (Fig. 6b). The mRNA expression of the *Slc2a1-5*, which encode GLUTs, were not altered with the GPR120 interventions (Fig. 6c). Taken together, these results indicated that GPR120 did not affect GLUT-mediated glucose uptake in DCs of FHF mice.

Glucose taken up by cells may undergo glycolysis, the pentose-phosphate pathway (PPP), tricarboxylic acid cycle (TCA), or glycogen synthesis. We observed that the protein levels of key enzymes regulating glucose metabolism, including glucose-6-phosphate dehydrogenase (G6PD, the key enzyme in the PPP), hexokinase 2 (HK2, the key enzyme in glycolysis), pyruvate dehydrogenase (PDH, the key enzyme in TCA), and lactate dehydrogenase (LDHA, a key enzyme in gluconeogenesis), were all increased during DC mature culture (Fig. 6d). To determine which glucose utilization pathway is regulated by GPR120 in DCs, we analyzed these enzymes during DC maturation and found that only HK2 was downregulated by TUG891 (Fig. 6e). The expression of HK2 was also elevated in GPR120^−/−^ DCs (Fig. 6f). Furthermore, when HK2 was inhibited by bromopyruvic acid, the promotion of DC generation and maturation by GPR120 deletion or AH7614/TUG891 was attenuated (Figs. 6g–i). While the anti-inflammatory cytokine levels including TGF-β and IL-10 were decreased in GPR120 deletion or AH7614-pretreated DCs, they were restored when glycolysis was suppressed (Fig. 6j). In addition, inhibition of glycolysis in GPR120-deficient or AH7614-pretreated DCs reduced the activation of CD4^+^ T cells (Fig. 6k), further indicating that GPR120 induced regulatory DCs by inhibiting glycolysis.

### GPR120 inhibited HIF-1α by ERK and AMPK pathway to induce regulatory DCs

**Fig. 7:**
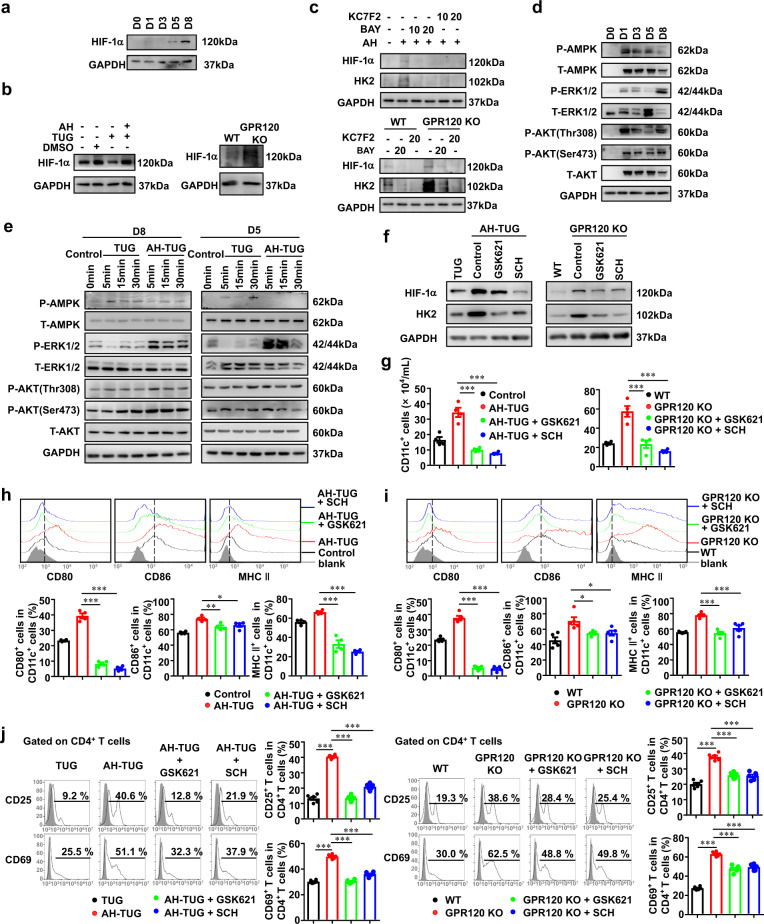
GPR120 inhibited HIF-1α expression to induce regulatory DCs mediated by the ERK and AMPK pathways. **a** The protein expression of HIF-1α in CD11c^+^ DCs was determined by immunoblot analysis during DC maturation. **b** BMCs from wild-type (WT) and GPR120^−/−^ (GPR120 KO) mice were isolated and cultured to induce maturation. DMSO, TUG891 (TUG, 20 μM) with or without AH7614 (AH, 20 μM) pretreatment was added to the medium of BMCs from WT mice every 2 days. LPS (0.1 μg/mL) was added on day 7. The protein expression of HIF-1α in CD11c^+^ DCs was determined by immunoblot analysis on day 8. **c** DCs were cultured to induce maturation for 8 days and supplemented with the HIF-1α inhibitor BAY 87-2243 (BAY, 10 μM or 20 μM). HIF-1α and HK2 levels were determined by immunoblot analysis. Another HIF-1α inhibitor KC7F2 (10 μM or 20 μM) was also used to confirm the result. **d** Levels of AMPK, ERK1/2, and AKT signaling pathways expression were determined by immunoblot analysis during DC maturation. **e** DCs were stimulated by TUG (20 μM) for 5 min, 15 min, and 30 min, with or without 1 h of pretreatment with 20 μM AH. Immunoblot analysis was performed to examine the AMPK, ERK1/2, and AKT signaling pathways on day 8 and day 5. **f**–**i** An AMPK activator (GSK621, 10 μM), the ERK1/2 inhibitor SCH772984 (SCH, 5 μM) was added to the culture medium of DCs pretreated with TUG (20 μM) plus AH (20 μM) and GPR120^−/−^ DCs for 12 h on day 8. **f** The protein expression of HIF-1α and HK2 in CD11c^+^ DCs was determined by immunoblot analysis. **g** Number of CD11c^+^ DCs was analyzed by flow cytometry. **h**, **i** The expression levels of CD80, CD86, and MHC II were analyzed by flow cytometry. **j** GSK621 (10 μM) or SCH (5 μM) was added into the medium of TUG (20 μM) with AH (20 μM) pretreated-WT DCs and GPR120^−/−^ DCs. CD4^+^ T cells were cocultured with DCs and T cell activation was measured by flow cytometry the next day. The results are representative of three to six independent experiments and presented as the mean ± SEM. Significant differences were analyzed by One-way ANOVA. *p < 0.05, **p < 0.01, ***p < 0.001.

We next examined the expression of HIF-1α, a critical transcription factor of HK2. The results revealed that HIF-1α was upregulated in DCs during maturation culture in vitro (Fig. 7a). Moreover, HIF-1α level was reduced by TUG891 but increased by AH7614 pre-treatment or GPR120 deletion (Fig. 7b). Next, we determined the role of HIF-1α in GPR120-regulated glycolysis by inhibiting HIF-1α using KC7F2 or BAY 87-2243. It was found that the HK2 upregulation by GPR120 antagonist or GPR120 deficiency was abolished with HIF-1α inhibition (Fig. 7c), suggesting that the upregulation of glycolysis in response to GPR120 inhibition or deletion was HIF-1α-dependent. AMPK, ERK1/2, and AKT respond to GPR120 activation [32, 33]. During DC maturation and activation, the phosphorylation of ERK1/2 and AKT was enhanced, and the phosphorylation of AMPK was decreased (Fig. 7d). The phosphorylation of ERK1/2 was markedly elevated in activated DCs at both day 8 and day 5 after AH7614 pretreatment, but was attenuated by TUG891 treatment (Fig. 7e). AMPK was activated by TUG891, while the phosphorylation level of AKT at Ser473 and Thr308 showed faint change (Fig. 7e). These results indicated that the ERK and AMPK pathways may participate in regulatory DCs regulation caused by GPR120 activation during FHF pathogenesis.

We next resorted to ERK1/2 inhibitor SCH772984 and AMPK activator GSK621 to determine whether GPR120 regulates HIF-1α through the ERK and AMPK pathways. It was found that HIF-1α and HK2 upregulation could be attenuated by ERK1/2 inhibition and AMPK activation, respectively, in TUG891/AH7614-treated DCs as well as in GPR120^−/−^ DCs (Fig. 7f). Furthermore, inhibition of ERK1/2 or activation of AMPK could also impair the generation of CD11c^+^ DCs and DC maturation (Figs. 7g–i). In addition, the activation of CD4^+^ T cells was greatly inhibited when the aberrant signaling caused by GPR120 antagonism or deletion was blocked in DCs (Fig. 7j).

### GPR120 activation correlated with the severity of FHF in patients

**Fig. 8:**
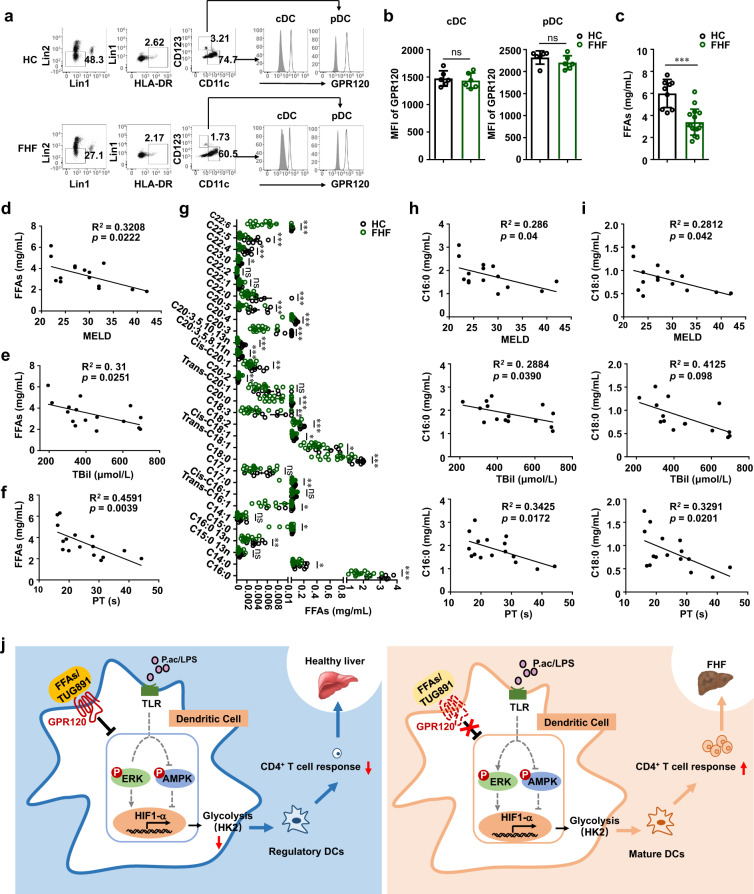
Plasma FFA was negatively correlated with prognostic scores of patients with fulminant hepatitis. **a** Human peripheral blood (PB) cells were collected from healthy controls (HC) and FHF patients (HC: *n* = 9, FHF: *n* = 16). Cells were stained for surface markers, permeabilized with the intracellular fixation and permeabilization buffer. Then cells were stained with anti-GPR120 antibody. Flow cytometry was used to measure GPR120 expression on conventional dendritic cells (cDCs) and plasmacytoid dendritic cells (pDCs) among the Lin1^−^Lin2^−^HLA-DR^+^ cell population in PBMCs from HC and FHF patients. Representative images were shown. **b** Mean fluorescence intensity (MFI) of GPR120 was shown (HC: *n* = 9, FHF: *n* = 16). **c**, **g** The levels of FFAs (C14–C22) in the plasma of HC and FHF patients (HC: *n* = 9, FHF: *n* = 16) were measured by Gas chromatography-mass spectrometry (GC–MS). **d** The correlation between plasma total FFAs (C14–C22) and the model for end-stage liver disease (MELD). **e** The correlation between total plasma (C14–C22) FFAs and total bilirubin (TBil). **f** The correlation between total plasma FFAs (C14–C22) and prothrombin time (PT). **h** The correlation between C16:0 and the MELD, TBil, or PT. **i** The correlation between C18:0 and the MELD, TBil, or PT. **j** The working model of GPR120 in protecting against FHF. During the pathogenesis of FHF, the levels of FFAs in the liver microenvironment were low. *P. acnes*/LPS activated the signaling pathway mediated by TLR in DCs, which promoted glycolysis by inducing the expression of HK2 on a HIF-1α-dependent manner. Supplement of FFAs or agonist promoted the activation of GPR120 and then impaired the glycolysis by regulating ERK and AMPK pathways, which further induced regulatory DCs in liver. Regulatory DCs inhibited liver-infiltrated pathogenic CD4^+^ T cell response, and finally suppressed the inflammation-induced acute liver injury. The results are presented as the mean ± SEM. Significant differences were analyzed by the Mann–Whitney *U* test (**b**, **c**, and **g**), Spearman’s rank correlation test (**d**, **e**, **f**, **h**, and **i**) *p < 0.05, **p < 0.01, ***p < 0.001, ns no significance.

To evaluate whether activation of GPR120 on DCs may be associated with the pathogenesis of FHF in human, we determined the GPR120 expression profile in human DCs. We observed that GPR120 was expressed in both cDCs and pDCs from healthy controls (HC) and FHF patients (Fig. 8a). Further comparison of the mean fluorescence intensity (MFI) of GPR120 between the two groups showed no significant difference (Fig. 8b). These results indicated that the expression of GPR120 could not indicate the progression of FHF.

We speculated that levels of the ligands of GPR120, such as saturated (C14–C18) and unsaturated (C16–C22) FFAs, may indicate the activation state of GPR120 and correlate with the severity of FHF. Thus, the levels of FFAs in the plasma of HC and FHF patients were analyzed. As shown in Fig. 8c, the amounts of total FFAs (C14–C22) in the plasma of FHF patients was significantly lower than those in HC. MELD is the most common predictor of the severity and prognosis of liver failure. At baseline, the total FFAs were negatively correlated with MELD (*P* < 0.05) (Fig. 8d), TBil (*P* < 0.05) (Fig. 8e) and prothrombin time (*P* < 0.01) (Fig. 8f). Analysis of individual FFAs indicated that the levels of many types of FFAs, especially C16:0 (palmitic) and C18:0 (stearic) in the plasma of FHF patients, were significantly lower than in HC (Fig. 8g). Likewise, the levels of C16:0 (palmitic) and C18:0 (stearic) were negatively correlated with disease severity (Figs. 8h–i). These results suggested that GPR120 activation was negatively correlated with the severity of FHF in patients.

## Discussion

Most studies have shown that abnormal G-protein-coupled receptor function during disease process arises either due to increased ligand production or increased receptor expression. Therefore, we used GPR120^−/−^ mice and GPR120 agonist TUG891 to determine the role of GPR120 in FHF mice. Our studies reveal that GPR120 plays a protective role in FHF by inducing regulatory DC generation. When activated by TUG891 or FFAs, GPR120 could downregulate HIF-1α in DCs by inhibiting ERK1/2 signaling and activating AMPK signaling, which leads to decreased HK2 expression and subsequently the inhibition of glycolysis. The induction of regulatory DCs due to the inhibition of glycolysis weakens T cell responses to ameliorate FHF.

DCs, one type of antigen-presenting cells recruited to the liver, play important roles in the progression of *P. acnes*-induced liver injury. We observed that DC activation was increased in the livers of GPR120^−/−^ mice and decreased by TUG891 treatment in the disease model, indicating that activation of GPR120 could regulate DC function. GPR120 activation and deletion had no effect on KC activation in *P. acnes*-induced liver injury. It was reported that in hepatic ischemia reperfusion injury and insulin resistance-associated obesity, GPR120 activation promotes M2 marker expression in macrophages, suggesting that GPR120 has distinct roles in regulating macrophages in different kinds of inflammatory diseases [14, 34]. Detailed analyses demonstrated that TUG891 induced a distinct DC population characterized by a CD11c^+^MHC II^low^CD80^low^CD86^low^ phenotype with potent regulatory function, which produced low levels of proinflammatory cytokines and were defective in stimulating activation of allogeneic T cells in MLR. Conventional mature DCs express high levels of MHC II and costimulatory molecules, produce large amounts of proinflammatory cytokines (e.g., TNF-α, IL-1β, and IL-12), have great ability to stimulate proliferation and expansion of allogeneic T cells in MLR. However, the immature DCs are characterized by low T-cell activation potential accompanied by low expression of MHC II and costimulatory molecules [35]. Upon inflammatory stimulation, immature DCs are able to acquire both phenotypic and functional properties of mature DCs [36, 37]. Notably, despite in vitro stimulation with GM-CSF plus TNF-α for 3 days, which has been shown to effectively induce maturation of conventional immature DCs, TUG891-treated DCs fail to up-regulate the expression of CD80 and CD86 but up-regulate the production of anti-inflammatory cytokines. Taken together, these observations suggest that TUG891-treated DCs resemble regulatory DCs, but are distinct from either immature or mature conventional DCs which can elicit primary T cell responses.

Glycolysis, the PPP, and glycogen synthesis are necessary for DC maturation and activation [30]. Recent reports have shown that GPR120 is involved in glucose and lipid metabolism [38, 39]. We observed that the expression of glycolysis-associated kinases HK2 was changed at the protein level in DCs with GPR120 interference. As a driver of glycolysis, HIF-1α regulates the transcription of a large number of target genes that are responsible for glycolysis. HIF-1α is associated with inducing hypoxia-related genes and repairing cellular oxygen homeostasis [40, 41]. It can promote angiogenesis, cell growth, and glucose metabolism in cancer cells [41, 42]. Additionally, HIF-1α is critical for the maturation of DCs and the activation of T cells [43]. We discovered that GPR120 activation inhibited glycolysis in DCs by attenuating the activity of HIF-1α, through the ERK and AMPK pathways. In addition, modulating the phosphorylation of ERK and AMPK, which are the conventional signaling pathways downstream of GPR120, could reverse the AH7614- and GPR120 deletion-induced increases in DC generation and activation.

GPR120 activation promotes ERK phosphorylation, but recent studies also report GPR120 inhibits ERK phosphorylation in adipocytes and human prostate cancer cells [44, 45]. TLR could recognize pathogen-associated molecular patterns such as LPS and *P. acnes*, and then activate ERK signaling during DC maturation [46]. On this basis, GPR120 activation inhibited ERK phosphorylation while AH7614 and GPR120 deletion improved ERK phosphorylation to regulate DC tolerance in our study. Although the phosphorylation of AKT at Thr308 and Ser473 barely changed when GPR120 was activated or blocked in DCs, inhibition of AKT phosphorylation could also weaken DC function. PI3K/AKT promotes cell survival by directly inhibiting proapoptotic proteins (such as Bad) or by inhibiting proapoptotic signals produced by transcription factors (such as Foxo) [47]. AKT can also promote cell proliferation [47, 48]. Thus, the effect of PI3K/AKT inhibition on DC may be due to increased DC apoptosis and/or inhibition of DC proliferation. In addition, whether the signal pathways have additive effects on DCs needs further research and could be new therapeutic targets in *P. acnes*-induced liver injury.

In summary, our study reveals that GPR120 plays a protective role in FHF by inhibiting glycolysis in DCs. When activated by TUG891, GPR120 downregulated the expression of HIF-1α by activating AMPK and suppressing ERK1/2 signaling, which further decreased HK2 expression in liver DCs. Glycolysis was then decreased mediated by GPR120-downregulated HK2, to induce regulatory DCs in mouse livers, which gave rise to the suppression of CD4^+^ T cell-mediated immune responses in FHF mice (Fig. 8j). Our study suggests that agonists or FFAs that target GPR120 are promising agents for FHF therapy.

## Supplementary information

Supporting Table 1

Related Manuscript File

Related Manuscript File

## Data Availability

The data sets generated during and/or analyzed during the current study are available from the corresponding author on reasonable request.
