# Supplementary material for: GPR120 induces regulatory dendritic cells by inhibiting HK2-dependent glycolysis to alleviate fulminant hepatic failure
Source: Cell Death Dis. 2021 Dec 16;13(1):1. doi: 10.1038/s41419-021-04394-0 (PMC8674251; doi:10.1038/s41419-021-04394-0)
Supplement: Supplementary file 1 — Supporting Table 1 [file 41419_2021_4394_MOESM1_ESM.docx]

**Supporting information**

**Table S1.** **Quantitative real-time PCR primers.**

| **Mouse primers** |  | **Sequence** |
| --- | --- | --- |
| *Slc2a1* | forward | 5’-CAGTTCGGCTATAACACTGGTG-3’ |
|  | reverse | 5’-GCCCCCGACAGAGAAGATG-3’ |
| *Slc2a2* | forward | 5’-TCAGAAGACAAGATCACCGGA-3’ |
|  | reverse | 5’-GCTGGTGTGACTGTAAGTGGG-3’ |
| *Slc2a3* | forward | 5’-ATGGGGACAACGAAGGTGAC-3’ |
|  | reverse | 5’-GTCTCAGGTGCATTGATGACTC-3’ |
| *Slc2a4* | forward | 5’-GTGACTGGAACACTGGTCCTA-3’ |
|  | reverse | 5’-CCAGCCACGTTGCATTGTAG-3’ |
| *Slc2a5* | forward | 5’-CCAATATGGGTACAACGTAGCTG-3’ |
|  | reverse | 5’-GCGTCAAGGTGAAGGACTCAATA-3’ |
| *Actb* | forward | 5’-GGCTGTATTCCCCTCCATCG-3’ |
|  | reverse | 5’-CCAGTTGGTAACAATGCCATGT-3’ |
