# Supplementary material for: GPR120 induces regulatory dendritic cells by inhibiting HK2-dependent glycolysis to alleviate fulminant hepatic failure
Source: Cell Death Dis. 2021 Dec 16;13(1):1. doi: 10.1038/s41419-021-04394-0 (PMC8674251; doi:10.1038/s41419-021-04394-0)
Supplement: Supplementary file 3 — Related Manuscript File [file 41419_2021_4394_MOESM3_ESM.pdf]

DECLARATION OF CONTRIBUTIONS TO ARTICLE

ADMC

Manuscript Number: CDDIS-21-0327

Journal Name: *Cell Death & Disease* (the 'Journal')

Proposed Title of the Contribution:

GPR120 induces regulatory dendritic cells by inhibiting HK2-dependent glycolysis to alleviate fulminant hepatic failure (the 'Contribution)

Author(s):

Hongshuang Yu, Wanlin Yang, Jiefang Huang, Xiang Miao, Bei Wang, Xiaohui Ren, Yuting Gu, Qiwei Wang, Xinyuan Ding, Xin Guo, Fengtao Qian, Yanyun Zhang, Huanbai Xu, Leizhen Zheng, Min Jin (the 'Authors')

For all *CDDis* articles, each person named as an author in the published version must be able to show he or she has contributed substantially to the article.

Authorship credit should be based on 1) substantial contributions to conception and design, acquisition of data, or analysis and interpretation of data; 2) drafting the article or revising it critically for important intellectual content; and 3) final approval of the version to be published. Authors should meet conditions 1, 2 and 3.

Any person who cannot be shown to have made a substantial contribution to the article cannot be listed as an author in the final version. The name of any person who is deemed to have made a minor contribution can, however, appear in the Acknowledgments section of the article.

Please complete the table below to indicate the contributions of all named authors to the manuscript.

Author Full Name:	Specification of Contribution to the Manuscript:
Hongshuang Yu	wrote and revised the manuscript, performed the experiments and analyzed the data, test clinical parameters
Wanlin Yang	wrote and revised the manuscript, performed the experiments and analyzed the data, collection of clinical parameters
Jiefang Huang	performed the experiments of dendritic cell function in vitro, design the experiments, revised the manuscript
Xiang Miao	directed flow cytometry staining, testing and data processing
Bei Wang	helped to perform the experiments, acquisition of data, or analysis
Xiaohui Ren	helped to perform the experiments, acquisition of data, or analysis
Yuting Gu	helped to supervise the project and analyzed the data
Qiwei Wang	helped to perform the experiments, acquisition of data
Xinyuan Ding	helped to supervise the project and analyzed the data
Xin Guo	helped to perform the experiments, acquisition of data
Fengtao Qian	helped to perform the experiments, acquisition of data
Yanyun Zhang	helped to supervise the project, designed study, and analyzed the data
Huanbai Xu	conceived and designed this study, analyzed the data

DECLARATION OF CONTRIBUTIONS TO ARTICLE

ADMC

Manuscript Number:	Journal Name:	
CDDIS-21-0327	*Cell Death & Disease*	(the 'Journal')

Proposed Title of the Contribution:

GPR120 induces regulatory dendritic cells by inhibiting HK2-dependent glycolysis to alleviate fulminant hepatic failure (the 'Contribution)

Author(s):

Hongshuang Yu, Wanlin Yang, Jiefang Huang, Xiang Miao, Bei Wang, Xiaohui Ren, Yuting Gu, Qiwei Wang, Xinyuan Ding, Xin Guo, Fengtao Qian, Yanyun Zhang, Huanbai Xu, Leizhen Zheng, Min Jin (the 'Authors')

For all *CDDis* articles, each person named as an author in the published version must be able to show he or she has contributed substantially to the article.

Authorship credit should be based on 1) substantial contributions to conception and design, acquisition of data, or analysis and interpretation of data; 2) drafting the article or revising it critically for important intellectual content; and 3) final approval of the version to be published. Authors should meet conditions 1, 2 and 3.

Any person who cannot be shown to have made a substantial contribution to the article cannot be listed as an author in the final version. The name of any person who is deemed to have made a minor contribution can, however, appear in the Acknowledgments section of the article.

Please complete the table below to indicate the contributions of all named authors to the manuscript.

Author Full Name:	Specification of Contribution to the Manuscript:
Leizhen Zheng	helped to supervise the project, designed study, and analyzed the data
Min Jin	conceived and designed this study, analyzed the data, and revised the manuscript.

Please complete the table below to indicate the contributions of all named authors to the figures.

Figure 1:

W. Y. and H. Y. built the animal model. W. Y. analysed the survival of mice, the weight of mice liver and spleen, the levels of ALT and AST in mice serum. H. Y. took the photos of mice liver and spleen, collected the liver tissues for HE staining. Y. Z. designed and conducted these experimental procedures.

Figure 2:

W. Y. and H. Y. built the animal model. W. Y., H. Y., J. H., F. Q. and B. W. isolated MNCs from mice livers and spleens. W. Y., H. Y. and X. M. prepared the staining protocol for FACS. H. Y. performed flow cytometry. W. Y. generated the data. M. J. designed and conducted these experimental procedures.

Figure 3:

W. Y. and H. Y. isolated MNCs from mice spleens. W. Y., H. Y. and X. M. prepared the staining protocol for FACS. H. Y. performed flow cytometry. W. Y. generated the data. H. X. designed and conducted these experimental procedures.

Figure 4:

W. Y. and H. Y. built the animal model. W. Y., H. Y., J. H., F. Q. and B. W. isolated MNCs from mice livers, spleens and peripheral blood. W. Y., H. Y. and X. M. prepared the staining protocol for FACS. H. Y. performed flow cytometry. H. X. designed and conducted these experimental procedures.

Figure 5:

W. Y. induced DC maturation in vitro and performed flow cytometry. H. Y. performed the mixed lymphocyte response and collected data by flow cytometry. H. Y. and J. H. performed the ELISA experiment and arranged the data. L. Z. designed and conducted these experimental procedures.

Figure 6:

W. Y. and H. Y. built the animal model. W. Y. performed the DC phagocytosis assay in vivo. Q. W. induced DC maturation in vitro and performed the DC phagocytosis assay. X. G. collected DC protein and RNA, performed q-PCR and WB, and collected the data. X. R. collected maturational DCs for marker staining, performed flow cytometry and collected the data. H. Y. performed the mixed lymphocyte response and collected data by flow cytometry. M. J. designed and conducted these experimental procedures.

Please complete the table below to indicate the contributions of all named authors to the figures.

Figure 7:

J. H. collected DC protein, performed WB, and collected the data. Y. G. collected maturational DCs for marker staining, performed flow cytometry and collected the data. X. D. performed the mixed lymphocyte response and collected data by flow cytometry. Y. Z. designed and conducted these experimental procedures.

Figure 8:

W. Y. collected Human peripheral blood (PB) cells HC and FHF patients. W. Y. performed flow cytometry, GC-MS and correlation analysis. L. Z. designed and conducted these experimental procedures.

Signed for and on behalf of the Author(s):	Print Name:	Date:
Jin Min	Min Jin	July 10, 2021
